# Polymer Lewis Base for Improving the Charge Transfer in Tin–Lead Mixed Perovskite Solar Cells

**DOI:** 10.3390/nano14050437

**Published:** 2024-02-27

**Authors:** Yanjun Xing, Zhiqiang Deng, Qiuxiang Wang, Jiaxing Xiong, Xiaohui Liu, Like Huang, Yuejin Zhu, Jing Zhang

**Affiliations:** 1 Department of Microelectronic Science and Engineering, Ningbo University, Ningbo 315211, China; 2111077040@nbu.edu.cn (Y.X.); zqdeng1@outlook.com (Z.D.); wangqiuxiang1102@outlook.com (Q.W.); jiaxingxiong@outlook.com (J.X.); liuxiaohui@nbu.edu.cn (X.L.); huanglike@nbu.edu.cn (L.H.); zhuyuejin@nbu.edu.cn (Y.Z.); 2 School of Information Engineering, College of Science and Technology, Ningbo University, Ningbo 315300, China

**Keywords:** Sn-Pb mixed perovskite, energy level regulation, metal coordination, defect passivation

## Abstract

The poor film stability of Sn-Pb mixed perovskite film and the mismatched interface energy levels pose significant challenges in enhancing the efficiency of tin–lead (Sn-Pb) mixed perovskite solar cells. In this study, polyvinylpyrrolidone (PVP) is introduced into the PVK perovskite precursor solution, effectively enhancing the overall stability of the film. This improvement is achieved through the formation of robust coordination bonds between the carbonyl (C=O) in the pyrrole ring and the undercoordinated Sn^II^ and Pb^II^, thereby facilitating the passivation of defects. Furthermore, the introduction of PVP inhibits the oxidation of tin (Sn), thereby enhancing the n-type characteristics of the perovskite film. This adjustment in the energy level of the PVK perovskite film proves instrumental in reducing interface energy loss, subsequently improving interface charge transfer and mitigating device recombination. Consequently, perovskite solar cells incorporating PVP achieve an outstanding champion power conversion efficiency (PCE) of 21.31%.

## 1. Introduction

Perovskite is a significant inorganic crystal structure commonly employed to characterize materials with the ABX_3_ arrangement. In this structure, the A site comprises MA^+^, FA^+^, and Cs^+^, the B site consists of metal ions (Pb^II^ or Sn^II^), and X represents non-metal ions, typically halide ions (I^−^, Cl^−^, or Br^−^). Perovskite solar cells have garnered considerable attention due to their relatively straightforward fabrication processes and excellent photovoltaic conversion performance. In these materials, organic compounds are commonly introduced to enhance material stability and processability. With their high efficiency and low production costs, perovskite solar cells have become a focal point in solar energy research. In recent years, the power conversion efficiency (PCE) of organic–inorganic hybrid perovskite solar cells (PSCs) has experienced rapid advancements, surpassing 26% [1] and approaching the efficiency levels of the best currently available silicon solar cells [1]. This underscores the promising application potential of perovskite solar cells [2]. However, the commercial utilization of pure lead (Pb)-based perovskites is still hindered by their toxicity [3]. Furthermore, the bandgap exceeding 1.5 eV in pure Pb-based PSCs constrains further progress in power conversion efficiency (PCE), as it falls outside the optimal bandgap range defined by the Shockley–Queisser (S-Q) limit [4]. To mitigate the toxicity of lead, effective substitution with tin (Sn) has emerged as a promising alternative. Sn has proven to be the most efficient replacement element. Doping perovskites with varying Sn content allows for the adjustment of the bandgap within the range of 1.2 to 1.4 eV, aiming to achieve the S-Q limit [5,6]. Additionally, breaking through the theoretical efficiency limit of individual cells has led to widespread interest in all-perovskite tandem solar cells. Continual development of Sn-Pb mixed PSCs as the top cell in tandem solar configurations is currently underway [7].

Despite Sn and Pb elements belonging to the same main group, they exhibit essential differences in their properties. In contrast to Pb, Sn is characterized by a deficiency in lanthanide contraction. This implies that the Sn element possesses higher atomic energy levels, lower electronegativity, and a smaller splitting between s and p orbitals. These distinctions contribute to the distinct behaviors and characteristics of Sn and Pb within the context of material science and electronic properties [8,9]. This leads to Sn elements having lower ionization energy, making them more prone to losing electrons from the 5 s orbital and exhibiting oxidation phenomena. The lower ionization energy of Sn implies a greater tendency for electron release, contributing to its reactivity and facilitating the occurrence of oxidation reactions [10]. From the perspective of perovskite films, the oxidation of Sn^II^ gives rise to the formation of a substantial quantity of vacancy defects (V_Sn_) and Sn^IV^ within the perovskite film. Additionally, the spontaneous transformation of Sn^IV^ to Sn^II^ induces the release of two holes into the valence band, thereby triggering p-type self-doping in the perovskite film. This phenomenon underscores the dynamic interplay between oxidation processes and the resulting impact on the electronic properties of the perovskite material [11]. This sequence of reactions ultimately culminates in pronounced non-radiative recombination and consequential degradation of the Sn-Pb mixed perovskite films. The intricate interplay of these reactions highlights their detrimental impact on the film’s stability and overall photovoltaic performance. Currently, a myriad of additives is incorporated into perovskite precursor solutions to enhance the stability of both the solution and the resulting film. Among these, reducing agents have proven to be particularly effective in inhibiting the oxidation of Sn^II^ by introducing functional groups with reducing capabilities. Examples of such reducing agents include dopamine cation (DAH^+^), caffeic acid (CA), hydrazine sulfate (HS), and others. The addition of these reducing agents to the perovskite precursor solution serves as a strategic approach to mitigate the oxidation of Sn^II^ and thereby enhance the overall stability of the perovskite film [12,13,14]. One effective method for enhancing film stability involves the incorporation of Lewis base additives, which form strong coordination bonds with Sn^II^ atoms, thereby suppressing their oxidation. Liu et al. successfully employed this approach to suppress the oxidation of Sn^II^ by introducing imidazole derivative (2-AD) as a coordination agent. The coordination effect between 2-AD and Sn^II^ atoms played a pivotal role in inhibiting the oxidative processes, contributing to the improved stability of the perovskite film [15]. Furthermore, Lewis bases exhibit the capability to passivate uncoordinated metal elements, thereby effectively diminishing the density of defect states within the material [16,17]. The pyrrole ring structure, commonly employed as a Lewis base, demonstrates effective coordination with metal atoms [18]. Notably, the addition of pyrrolidone to the precursor solution of pure Pb-based perovskite has been observed, contributing to both the enhanced stability of the film and the passivation of defects. This underscores the multifunctional role of Lewis bases in mitigating material defects and improving overall film performance [19,20].

In this study, polyvinylpyrrolidone (PVP) is incorporated into the Sn-Pb mixed perovskite precursor solution. On the one hand, the lone pair of electrons of the carbonyl (C=O) in PVP effectively form strong coordination bonds with metal atoms, thereby reducing the density of defect states. The inhibition of Sn^II^ oxidation is attributed to the robust interaction between C=O and Sn atoms, coupled with the weak reducibility of PVP. On the other hand, the p-type characteristics of the Sn-Pb perovskite film are mitigated as a result of the inhibited Sn^II^ oxidation. This adjustment effectively tunes the Fermi level of the perovskite and optimizes the energy level structure between the perovskite and PEDOT:PSS. The improved energy level structure facilitates hole transfer at the interface, thereby suppressing interface charge recombination. Ultimately, these enhancements contribute to the achievement of a Sn-Pb mixed perovskite solar cell with a power conversion efficiency (PCE) of 21.31%, coupled with enhanced stability in both air and glove boxes.

## 2. Results and Discussion

**Figure 1:**
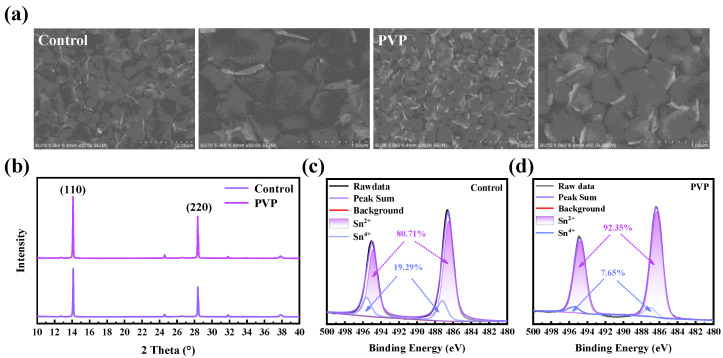
(**a**) The SEM images of control and PVP film; (**b**) the XRD of control and PVP film; the Sn 3d XPS fit spectra of (**c**) control and (**d**) PVP.

To assess the impact of polymer addition on film quality, the morphology and crystallinity of the film are first characterized. Scanning electron microscopy (SEM) is employed for observing the film morphology, providing detailed insights into the surface structure and texture. Through SEM analysis, the influence of the polymer addition on the film’s physical attributes and overall quality can be effectively discerned. As shown in Figure 1a, the morphology of the film with PVP, when compared to the film without PVP (which will subsequently be referred to as the control film), exhibits no significant changes. This observation indicates that the addition of polymers does not induce any discernible damage to the film’s morphology. The similarity in morphology between the film with PVP and the control film suggests that the polymer inclusion has a negligible impact on the overall structural integrity and appearance of the film. Furthermore, X-ray diffraction (XRD) is employed to assess the crystallinity of the film, as illustrated in Figure 1b. The introduction of PVP leads to a notable enhancement in the crystallinity of the film. We conducted a detailed analysis of the peak signals corresponding to the 110 and 220 crystal planes in the XRD spectrum, meticulously calculating their respective half-widths. In contrast to the control film, noteworthy improvements were observed in the PVP film, with the half-widths of the (110) and (220) crystal plane signals decreasing from 0.0795 and 0.116 to 0.0784 and 0.074, respectively. This reduction in half-width suggests a refinement in the crystal structure of the PVP film. Moreover, the peak intensity exhibited a notable enhancement compared to the control film, pointing towards an overall improvement in crystallinity. These findings collectively indicate a more well-defined and improved crystal lattice structure in the PVP film. Furthermore, as depicted in Figure S1, it is noteworthy that the peak signal associated with the (110) crystal plane in the XRD did not display a discernible shift. This observation implies that the introduction of PVP did not lead to a more pronounced strain phenomenon. The absence of significant peak shifts suggests that the incorporation of PVP did not exert a substantial impact on the structural integrity or strain state of the material, as evidenced by the XRD analysis. Despite the absence of observable changes in morphology, the XRD results indicate a discernible improvement in the ordered arrangement of crystalline structures within the film after the addition of PVP. This suggests that the presence of PVP positively influences the crystalline characteristics of the film, contributing to its overall structural quality. In fact, PVP serves as a reductive agent when utilized for the preparation of Ag and Au by reducing AgNO_3_ and HAuCl_4_ precursors. The reducing capability of PVP can be attributed to the nitrogen atom within the five-membered heterocyclic ring. This nitrogen atom plays a crucial role in the reduction process, contributing to the effective reduction of metal precursors and highlighting the versatile reductive properties of PVP in various applications [21]. Hence, X-ray photoelectron spectroscopy (XPS) is employed to analyze the content of Sn^II^ and Sn^IV^ in the films, providing valuable insights into their chemical composition [22]. As illustrated in Figure 1c,d, in comparison to the control film, the content of Sn^IV^ in the PVP film is significantly reduced from 19.26% to 7.65%. This reduction, below that observed in the control film, suggests an enhanced antioxidant capacity of the PVP film. Importantly, the XPS spectrum, focused on surface interactions with oxygen, does not exhibit any signal peak indicative of the presence of Sn^0^. The decrease in Sn^IV^ content is attributed to the robust interaction between PVP and Sn, coupled with the weak reducibility of PVP. The stronger binding between PVP and Sn atoms renders the oxidation of Sn^II^ more challenging, highlighting the film’s improved resistance to oxidation processes.

**Figure 2:**
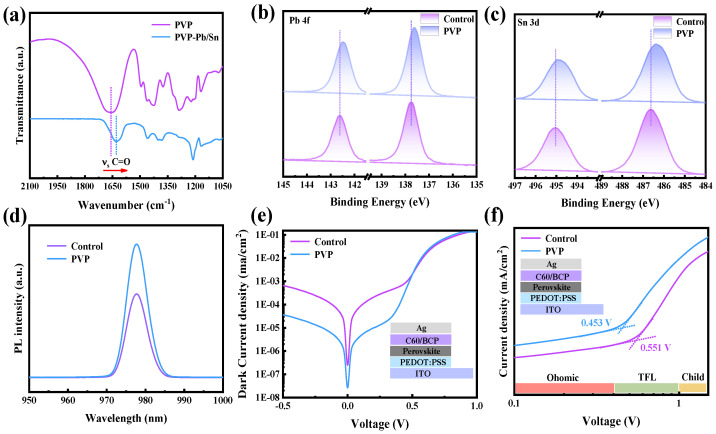
(**a**) The FTIR of PVP and PVP-Sn/Pb film; the XPS of (**b**) Pb 4f and (**c**) Sn 3d; (**d**) the dark current of control and PVP devices; (**e**) the PL of control and PVP film; (**f**) the SCLC of control and PVP devices.

The carbonyl groups (C=O) present in PVP function as Lewis base groups, effectively engaging with uncoordinated metal elements through the lone pair of electrons associated with the oxygen atom in C=O. This interaction is crucial for the formation of strong coordination bonds, highlighting the Lewis basicity of PVP and its capability to participate in coordination chemistry with metal elements in the system. To further investigate the interaction between C=O in PVP and metal elements, Fourier transform infrared spectroscopy (FTIR) is employed to monitor changes in wavenumber, providing insights into the occurrence of chemical reactions. As depicted in Figure 2a, observable shifts in the wavenumber of C=O are noted. Specifically, the wavenumber of C=O in PVP undergoes a shift from 1657.7 cm^−1^ to a lower value of 1627.6 cm^−1^, indicating the occurrence of a chemical interaction between C=O and metal atoms. This spectral change serves as evidence for the formation of coordination bonds between the carbonyl groups in PVP and the metal elements, affirming the strong interaction observed in the system. Nevertheless, the specific nature of the chemical reaction remains unclear. To further elucidate the types of chemical interactions between C=O and metal atoms, X-ray photoelectron spectroscopy (XPS) is employed to observe changes in the binding energy of metal atoms. As depicted in Figure 2b and c, a noticeable shift towards lower binding energy is observed in the XPS spectra of Sn 3d and Pb 4f. This shift in binding energy suggests a chemical interaction stemming from the interaction between metal atoms and Lewis base groups providing electrons [23]. It is thereby confirmed that PVP can effectively passivate metal atoms coordinated with a lone pair of electrons in C=O [24]. This strong interaction between C=O and metal atoms serves a dual purpose: firstly, enhancing the material’s resistance to oxygen; secondly, effectively reducing the density of defect states by coordinating with uncoordinated metal atoms. This dual mechanism further underscores the advantageous role of PVP in enhancing both the stability and electronic properties of the perovskite film.

Hence, the steady-state photoluminescence (PL) spectrum is measured to assess the intensity of the emission peak arising from the photoluminescence effect. As depicted in Figure 2d, in comparison to the control film, the PVP film exhibits a stronger emission peak intensity. This observation suggests a lower incidence of non-radiative recombination in the PVP film. The increased emission peak intensity in the presence of PVP indicates improved radiative recombination processes, emphasizing the beneficial impact of PVP in reducing non-radiative losses within the perovskite film [25]. Furthermore, the dark current of devices is measured to qualitatively analyze changes in defect density. In comparison to the control devices, the PVP devices exhibit a lower dark current density, as illustrated in Figure 2e. Dark current in the film primarily results from the movement of charge carriers induced by defect states when the device is not illuminated. A lower dark current density indicates a reduction in the density of defect states, signifying that the PVP device possesses a lower defect density. This outcome underscores the efficacy of PVP in mitigating defect-related issues within the perovskite film. Additionally, we corroborated the dark current results by examining the photoluminescence effect of the perovskite films. Furthermore, transient photo-voltage decay (TPV) measurements were conducted to observe the photo-voltage decay of the devices. As depicted in Figure S2, the PVP devices exhibit a slower decay time of photo-voltage, indicative of fewer non-radiative recombination events. The TPV results align with the findings from dark current and PL measurements. To further analyze defect density, space-charge-limited current (SCLC) is employed. As illustrated in Figure 2f, the trap-filled limit voltage (VTFL) of the device decreases from 0.551 V to 0.453 V. This reduction in VTFL signifies a decrease in trap density within the PVP device, affirming the consistent trend observed across multiple analyses. These comprehensive results collectively underscore the positive impact of PVP in mitigating non-radiative recombination and reducing defect density within the perovskite film. Utilizing the formula:

$$\eta_{trap}=\frac{{2ɛ}_{o}{ɛ}_{r}V_{TFL}}{ed^{2}}$$

where $\eta_{trap}$, ɛ*_o_*, ɛ*_r_*, *e*, and *d* are the trap states density, vacuum permittivity, dielectric constant, elementary charge, and film thickness. The defect density can be calculated. The defect density of PVP devices is determined to be 3.51 × 10^15^ cm^−3^, significantly lower than that of the control device (4.26 × 10^15^ cm^−3^). This reduction in defect density is one of the key factors contributing to the higher open circuit voltage observed in PVP devices, highlighting the positive influence of PVP in minimizing defects within the perovskite film.

**Figure 3:**
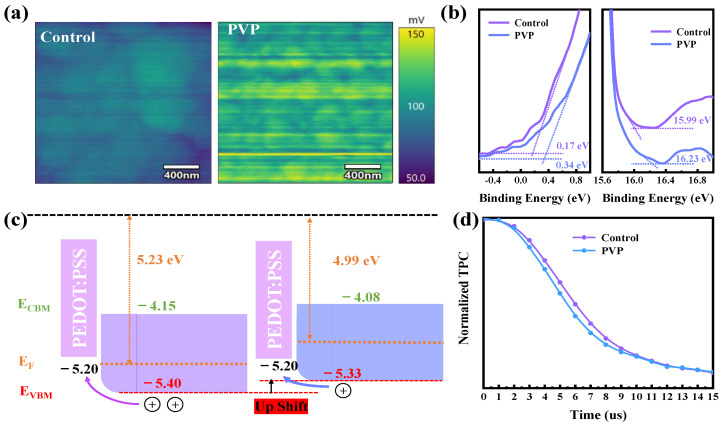
(**a**) KPFM images of control and PVP film; (**b**) the UPS of control and PVP; (**c**) the energy level structure of control and PVP film; (**d**) the TPC of control and PVP devices.

In addition to the impact of non-radiative recombination within the film, the suboptimal energy level structure between the perovskite and PEDOT:PSS serves as another source of open circuit voltage loss (Vloss). The inefficient alignment of energy levels between these two materials contributes to reduced voltage output in the device. Addressing and optimizing this energy level mismatch is crucial for enhancing the overall performance and efficiency of the perovskite solar cell [26,27]. The pronounced p-type doping resulting from the oxidation of Sn^II^ at the valence band top induces a significant deviation between the perovskite and PEDOT:PSS in terms of their energy levels. With the inhibition of Sn^II^ oxidation in the film, the p-type characteristics of the perovskite film have been mitigated to a certain extent following the addition of PVP. This improvement in p-type characteristics contributes to a more favorable alignment of energy levels between the perovskite and PEDOT:PSS, addressing the energy level mismatch and reducing the associated open circuit voltage loss. Kelvin probe force microscopy (KPFM) is employed to analyze changes in surface potential. As illustrated in Figure 3a, the surface potential of the PVP film increases, indicating a reduction in the work function of the perovskite film and an upshift in the Fermi energy after the addition of PVP. The inhibition of Sn^II^ oxidation in the PVP film results in only a minimal amount of Sn^IV^ releasing holes toward the valence band. This phenomenon leads to a gradual shift in the film’s characteristics from p-type to n-type due to the reduction in hole density. The observed changes in surface potential further emphasize the influence of PVP in modulating the electronic properties of the perovskite film.

For a more precise analysis of the Fermi level position and semiconductor characteristics, ultraviolet photoelectron spectroscopy (UPS) measurements are conducted. As depicted in Figure 3b, it is observed that the binding energy of the cut-off edge in the PVP film increases. This signifies a decrease in the work function (WF), leading to an upward shift in the Fermi level. The observed changes in the UPS results provide additional confirmation of the impact of PVP on the electronic structure of the perovskite film, contributing to a more comprehensive understanding of the material’s semiconductor properties. The reduction in the work function (WF) from 5.23 eV to 4.99 eV aligns consistently with the KPFM results. To further validate the observed trend of semiconductor characteristics transitioning from p-type to n-type in the Sn-Pb perovskite film after the addition of PVP, the starting edge is analyzed to ascertain the distance between the Fermi level and the valence band top. In comparison with the control, the distance increases from 0.17 eV to 0.34 eV in the PVP film, indicating a weaker p-type characteristic compared to the control film. This additional analysis strengthens the evidence supporting the shift in semiconductor properties induced by the incorporation of PVP into the perovskite film. The examination of the energy level structure between the Sn-Pb mixed perovskite film and PEDOT:PSS provides insights into the transfer performance. As illustrated in Figure 3c, the valence band top of the PVP film is found to be closer to the valence band top of PEDOT:PSS compared to the control film. This suggests that the PVP film exhibits more favorable hole transfer performance and lower energy loss. To further emphasize the excellent charge transfer capabilities of the devices, transient photocurrent decay (TPC) is measured to observe the decay of photocurrent. The decrease in TPC response from 7.16 μs to 6.55 μs, as shown in Figure 3d, indicates that PVP devices demonstrate enhanced transfer performance attributed to a more favorable energy level structure. This improved alignment contributes to efficient charge transfer and reduced losses in the overall energy conversion process.

**Figure 4:**
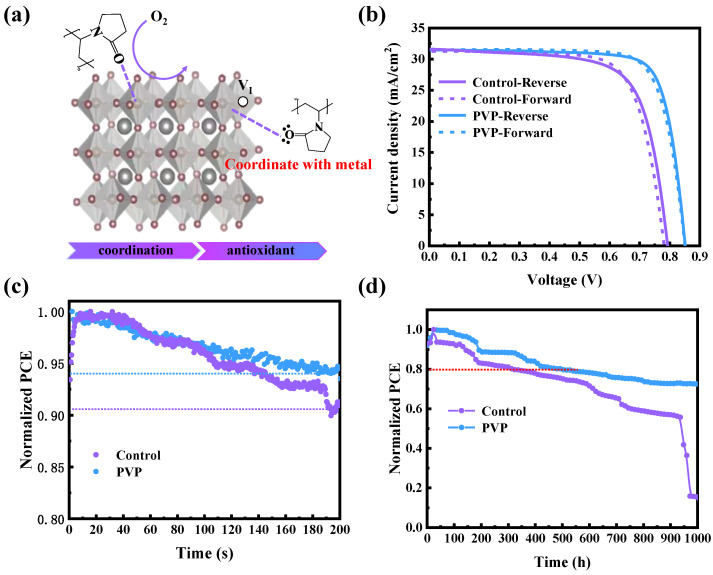
(**a**) The schematic illustration of PVP’s function in Sn-Pb perovskite film; (**b**) the J-V forward and reverse scanning curve of control and PVP devices; (**c**) the MPP of control and PVP devices; (**d**) the long-term stability of control and PVP devices.

As depicted in Figure 4a, the incorporation of PVP effectively imparts resistance to oxygen and diminishes uncoordinated metal elements through coordination. Consequently, as illustrated in Figure 4b, the champion power conversion efficiency (PCE) of 21.31% is achieved, which is attributed to the effective passivation of defects and the regulation of the energy level structure. Additionally, the external quantum efficiencies (EQE) of the devices are meticulously measured. As illustrated in Figure S3, the integral short-circuit current densities (Jsc) closely align with the measured Jsc values in the J-V curves. This concordance between EQE and J-V curve underscores the reliability and accuracy of the experimental measurements, providing robust confirmation of the device’s performance. In the final stage, the stability of the device is characterized, with a focus on both long-term stability and light stability. As illustrated in Figure 4d, devices incorporating PVP maintain 80% of their initial power conversion efficiency (PCE) after 1000 h, while the original device experiences rapid decay after 600 h. This underscores the significantly improved long-term stability achieved through the addition of PVP. Furthermore, the photostability of the device is assessed by measuring the maximum power point (MPP) to reflect its resistance to light-induced degradation, as shown in Figure 4c. In comparison with the control device, the PVP device exhibits superior photostability attributed to the inhibition of Sn^II^ oxidation. This enhanced stability under prolonged exposure to light further reinforces the positive impact of PVP on the durability and robustness of the perovskite solar cell.

## 3. Conclusions

In conclusion, our study demonstrates the robust coordination capability and defect passivation ability of polyvinylpyrrolidone. We have elucidated that the coordination mechanism is intricately connected to the carbonyl functional group in polyvinylpyrrolidone. Simultaneously, we have successfully adjusted the energy level structure by suppressing the characteristics of p-type semiconductors. Consequently, the devices incorporating polyvinylpyrrolidone exhibit improved power conversion efficiency (PCE) and long-term stability, attributed to the reduction in defect state density and optimization of the energy level structure. This work underscores the promising role of polyvinylpyrrolidone in enhancing the performance and stability of Sn-Pb mixed perovskite solar cells, offering valuable insights for future advancements in perovskite solar cell technology.

## 4. Experimental Section

### 4.1. Materials 

Unless otherwise stated, all chemicals and materials are purchased and used on receipt. Lead iodide (PbI_2_), PEDOT:PSS (Al 4083), methylammonium iodide (MAI), Formamidinium iodide (FAI), C_60_, and 2,9- dimethyl-4,7-diphenyl-1,10-phenanthroline (BCP) purchased from Xi’an Polymer Light Technology Corp (CN). The tin (II) iodide (SnI_2_), tin (II) fluoride (SnF_2_), and guanidine thiocyanate (PbSCN), polyvinylpyrrolidone (PVP), Sn powder are purchased from Aladdin (CN).

### 4.2. Materials Preparation

The method for preparing PEDOT:PSS solution involves mixing deionized water and PEDOT:PSS in a volumetric ratio of 3:1. Prior to utilization, the solution should be filtered through a 0.22 μm polyvinylidene fluoride (PVDF) filter. Preparation of FASnI_3_ perovskite precursor solution: Weigh 5 mg of Tin (Sn) metal powder, 7.8 mg of SnF_2_, 172 mg of FAI, and 372 mg of SnI_2_. Thoroughly combine these components, then add 424 μL of dimethylformamide (DMF) and 212 μL of dimethyl sulfoxide (DMSO) to facilitate the dissolution of the powder. This process yields a concentrated Sn solution with a concentration of 1.6 mmol/mL. To create a Sn solution incorporating PVP, introduce a 1% molar concentration of PVP powder into the above solution. The preparation method for MAPbI_3_ Perovskite Precursor Solution involves the careful weighing of 13.8 mg of PbSCN, 159 mg of MAI, and 461 mg of PbI_2_. Combine these components thoroughly, then add 565 μL of DMF and 71 μL of DMSO to dissolve the powder, resulting in a Pb solution with a concentration of 1.6 mmol/mL. Combine the two solutions in a 1:1 volume ratio to produce a tin–lead mixed perovskite precursor solution with a concentration of 1.6 mmol/mL. 

### 4.3. Fabrication of the Devices

Following chemical etching, perform sequential cleaning of the FTO glass substrate with detergent solution, deionized water, and alcohol. Subsequently, spin-coat the FTO substrate with a PEDOT:PSS solution (diluted with 3 mL deionized water per 1 mL PEDOT:PSA) at 4000 rpm for 40 s, followed by annealing at 150 °C for 15 min. Next, within a nitrogen glovebox, spin-coat the PEDOT:PSS layer with a perovskite solution at 1000 rpm for 5 s and 4000 rpm for 30 s (perovskite concentration: 1.4 mmol/mL). After initiating the second step, dispense 180 µL chlorobenzene onto the spinning substrate in the final 20 s of the program. Subsequently, anneal the substrate on a hot plate at 100 °C for 10 min. Preparation of the electron transport layer and metal electrodes involves vacuum deposition of C60 and BCP layers at a rate of 0.1 Å/s. The thickness of the C60 layer is 21 nm, and the BCP layer is 6 nm. Finally, a 70 nm thick Ag electrode is deposited by forward vacuum evaporation at a rate of 0.7 Å/s.

## Data Availability

The data presented in this study are available on request from the corresponding author.

## Supplementary Materials

The following supporting information can be downloaded at https://www.mdpi.com/article/10.3390/nano14050437/s1, Figure S1: The TPV of control and PVP devices; Figure S2. The EQE of control and PVP devices; Table S1: The photovoltaic performance parameters of devices.
