# Supplementary material for: Polymer Lewis Base for Improving the Charge Transfer in Tin–Lead Mixed Perovskite Solar Cells
Source: Nanomaterials (Basel). 2024 Feb 27;14(5):437. doi: 10.3390/nano14050437 (PMC10935037; doi:10.3390/nano14050437)
Supplement: Supplementary file 1 [file nanomaterials-14-00437-s001.zip › nanomaterials-2860362-supplementary.pdf]

nanomaterials

Supplementary Materials

Polymer Lewis Base for Improving the Charge Transfer in Tin–Lead Mixed Perovskite Solar Cells

Yanjun Xing [1], Zhiqiang Deng [1], Qiuxiang Wang [1], Jiaxing Xiong [1], Xiaohui Liu [1], Like Huang [1], Yuejin Zhu [1,2] and Jing Zhang [1,*]

1 Department of Microelectronic Science and Engineering, Ningbo University, Ningbo 315211, China; 2111077040@nbu.edu.cn (Y.X.); zqdeng1@outlook.com (Z.D.); wangqiuxiang1102@outlook.com (Q.W.); jiaxingxiong@outlook.com (J.X.); liuxiaohui@nbu.edu.cn (X.L.); huanglike@nbu.edu.cn (L.H.); zhuyuejin@nbu.edu.cn (Y.Z.)

2 School of Information Engineering, College of Science and Technology, Ningbo University, Ningbo 315300, China

* Correspondence: zhangjing@nbu.edu.cn.

Experimental Methods

Characterizations: Atomic force microscopy (AFM) is conducted on Veeco (America) and Kelvin probe force microscopy (KPFM) is used to detect the contact potential to reveal the fermi level and ion migration properties of the film under the bias applied normally to the film.

The surface morphology of the films and the cross-section morphology of the devices are tested by a Hitachi SU-70 scanning electron microscope (SEM). X-ray diffraction (XRD) patterns of films are tested by a Bruker D8 advanced instrument and using Cu Kα as radiation (λ=6.162Å) at a scan rate of 4°min^{-1} and diffraction angle range from 10° to 40°. The absorption spectra of the film on the glass substrate are measured by UV-visible spectrophotometer (Agilent, USA).

FTIR spectroscopy are taken with FTIR spectrometer instrument (Thermo, Nicolet 6700). The binding energies of the elements in PVK are tested by X-ray photoelectron spectroscopy (XPS, Shimadzu, Japan) using Al Kα radiation. Au is used to calibrating the energy state of the spectroscopy before measurement. The ultraviolet photoelectron spectroscopy (UPS) pattern is detected by the Axis Ultra DLD, and using the He I (21.22 eV) emission line. The steady-state fluorescence (PL) spectra of the films are measured based on the glass by a fluorescence spectrophotometer (Agilent, USA), with an excitation wavelength of 532 nm. The SCLC measurements and dark *J-V* curves are measured by the Keithley 4200 under condition.

The J-V characteristic curves are tested by the Keithley 4200 meter and the sunlight simulator (Newport, 91192A, AM 1.5, 1 sun) whose light intensity is calibrated by a standard silicon solar cell. The solar cells are masked with a black aperture to define the active area of 0.095 cm^2. The EQE is tested by Newport EQE system and wavelength range from 300 to 1100 nm. The transient photo-current (TPC) decay and transient photo-voltage (TPV) decay are recorded by an electrochemical workstation (Zahner, Germany) with a white light LED supplied 80 mWcm^{-2} light intensity to excite the perovskite solar cells. EIS is also tested by an electrochemical workstation at a bias voltage of 0.6V.

Parameter setting of the UPS spectrum peak in Sn 3d region: The binding energy positions for Sn^{IV} are set at 487 eV and 495.5eV, while for Sn^{II}, they are set at 486.3eV and 494.8eV. The The half-peak widths are uniformly configured at 1.4, the Lorentzian-Gaussian is 10%.

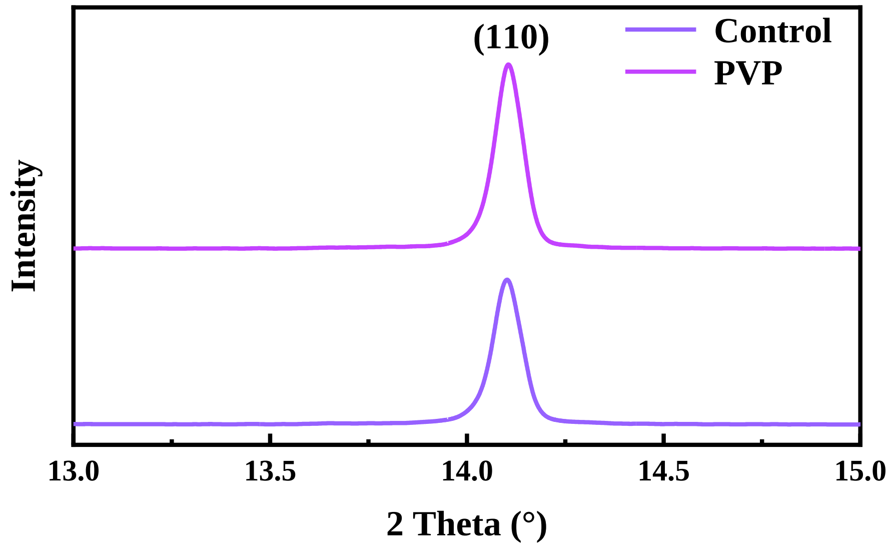

Figure S1. The XRD of control and PVP film.

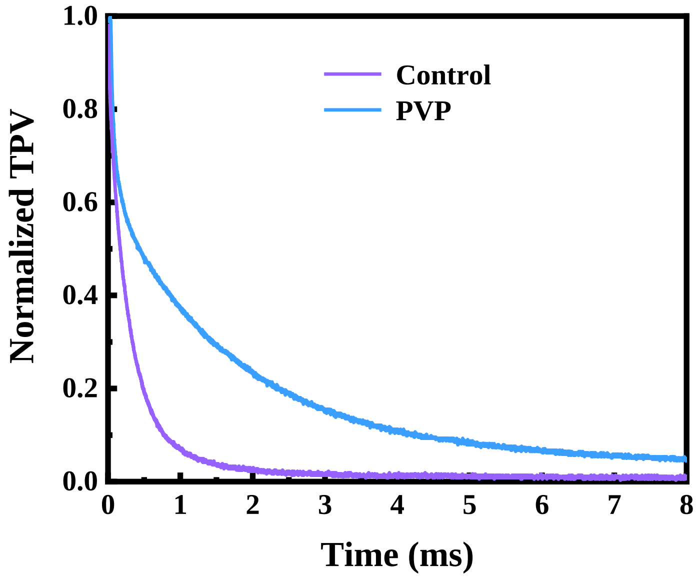

Figure S2. The TPV of control and PVP devices.

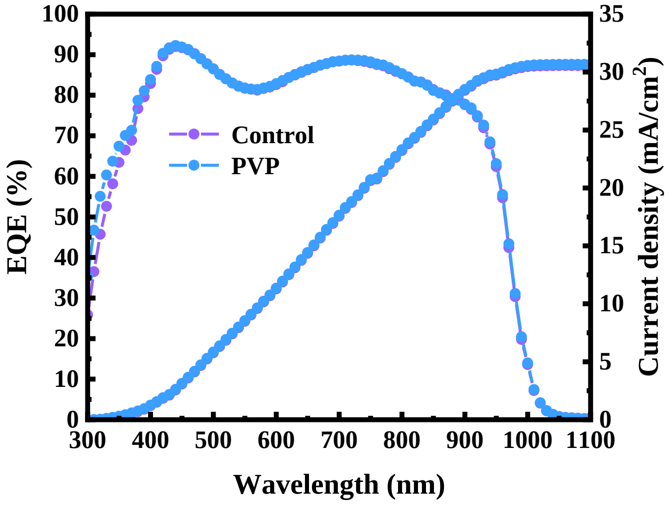

Figure S3. The EQE of control and PVP devices.

Table S1. The photovoltaic performance parameters of devices.

	Voc (V)	Jsc (mA/cm2)	FF (%)	PCE (%)
**Control-Reverse**	0.797	31.82	70.5	17.88
**Control-Forward**	0.784	31.44	73.0	17.99
**PVP-Reverse**	0.855	31.63	78.8	21.31
**PVP-Forward**	0.854	31.45	78.3	21.03
